# Duplication and Diversification of Dipteran Argonaute Genes, and the Evolutionary Divergence of Piwi and Aubergine

**DOI:** 10.1093/gbe/evw018

**Published:** 2016-02-11

**Authors:** Samuel H. Lewis, Heli Salmela, Darren J. Obbard

**Affiliations:** 1 Institute of Evolutionary Biology, University of Edinburgh, United Kingdom; 2 Department of Biosciences, Centre of Excellence in Biological Interactions, University of Helsinki, Helsinki, Finland; 3 Centre for Immunity, Infection and Evolution, University of Edinburgh, United Kingdom; 4 Present Address: Department of Genetics, University of Cambridge, Downing Street, Cambridge, CB2 3EH

**Keywords:** RNAi, Argonaute, Piwi, gene duplication, Diptera

## Abstract

Genetic studies of *Drosophila melanogaster* have provided a paradigm for RNA interference (RNAi) in arthropods, in which the microRNA and antiviral pathways are each mediated by a single Argonaute (Ago1 and Ago2) and germline suppression of transposable elements is mediated by a trio of Piwi-subfamily Argonaute proteins (Ago3, Aub, and Piwi). Without a suitable evolutionary context, deviations from this can be interpreted as derived or idiosyncratic. Here we analyze the evolution of Argonaute genes across the genomes and transcriptomes of 86 Dipteran species, showing that variation in copy number can occur rapidly, and that there is constant flux in some RNAi mechanisms. The lability of the RNAi pathways is illustrated by the divergence of Aub and Piwi (182–156 Ma), independent origins of multiple Piwi-family genes in *Aedes* mosquitoes (less than 25Ma), and the recent duplications of Ago2 and Ago3 in the tsetse fly *Glossina morsitans*. In each case the tissue specificity of these genes has altered, suggesting functional divergence or innovation, and consistent with the action of dynamic selection pressures across the Argonaute gene family. We find there are large differences in evolutionary rates and gene turnover between pathways, and that paralogs of Ago2, Ago3, and Piwi/Aub show contrasting rates of evolution after duplication. This suggests that Argonautes undergo frequent evolutionary expansions that facilitate functional divergence.

## Introduction

Argonaute genes of the Ago and Piwi subfamilies mediate a broad range of processes from development to antiviral immunity, and are found in almost all eukaryotes (Cerutti and Casas-Mollano 2006). They constitute an ancient gene family that was present in the common ancestor of extant prokaryotes and eukaryotes (reviewed in Swarts et al. 2014), and which diverged into Ago and Piwi subfamilies early in eukaryotic evolution (Cerutti and Casas-Mollano 2006; Mukherjee et al. 2013). The Argonautes are effectors in the RNA interference (RNAi)-related pathways, which can be broadly defined as a system of nucleic acid manipulation through complementary base pairing between small RNA (sRNA) guides and long nucleic acid targets. Each sRNA is loaded into an Argonaute protein, which it guides to a target nucleic acid, resulting in cleavage or translational inhibition of the target (reviewed in Sarkies and Miska 2014). Three broad classes of sRNA can be defined based on their sizes and interactors (reviewed in Kim et al. 2009): short interfering RNAs (siRNAs) are ∼21–24 nt long and are produced from viruses, transposable elements (TEs), and some long double-stranded RNA (dsRNA) products in the soma; microRNAs (miRNAs) are generally ∼22–23 nt long and are derived from host-encoded hairpin loops; and Piwi-interacting RNAs (piRNAs) are 24–29 nt long, derived largely from intergenic repetitive elements (e.g., TEs) in the germline, and exclusively bind Piwi-subfamily Argonaute proteins.

RNAi is well studied in *Arabidopsis thaliana*, where the Argonaute gene was first identified (Bohmert et al. 1998), and in the nematode *Caenorhabditis elegans*, where the RNAi mechanism was first characterized (Fire et al. 1998). Subsequent studies have reported Argonautes with diverse functions and differences in copy number across different eukaryotic clades (Mukherjee et al. 2013), illustrating that RNAi pathways have a dynamic evolutionary history. For example, in plants RNAi-mediated suppression of TEs is directed by shorter sRNAs than in animals, and is mediated by Agos not Piwis (which they lack completely; Cerutti and Casas-Mollano 2006; reviewed in Parent et al. 2012). Differences in Argonaute copy number and function are also found in the animals. In the protostomes, the planarian *Schmidtea mediterranea* has nine Piwi homologs (Palakodeti et al. 2008), two of which (smedwi-2 and smedwi-3) play vital roles in regeneration by facilitating the differentiation of pluripotent neoblasts (Reddien et al. 2005; Palakodeti et al. 2008). In contrast, Piwi and their associated piRNAs have been lost independently in several lineages of nematodes, with TE suppression carried out instead by DNA methylation mediated by RNA-dependent RNA polymerase and Dicer (Sarkies et al. 2015). Interestingly, this loss of Piwi has been accompanied by a massive expansion of other Argonaute genes in nematodes, with *Ca. elegans* encoding 25 Argonautes, 18 of which fall into the divergent worm-specific Ago (WAGO) clade: These associate with a novel class of sRNA (22G-RNAs) and carry out derived functions such as epigenetic memory formation (reviewed in Buck and Blaxter 2013).

Recent genome sequences and experimental data from isolated taxa have also revealed numerous arthropods with duplicates of Argonautes, some of which have novel and divergent functions. For example, the tick *Ixodes scapularis* has three Ago2 paralogs, only two of which appear to function in antiviral defense (Schnettler et al. 2014). Larger expansions are seen in the aphid *Acyrthosiphon pisum*, which has two paralogs of Ago3 and eight paralogs of Piwi, some of which are expressed in the soma (in contrast to *Drosophila melanogaster*, where they are predominantly germline specific; Lu et al. 2011). Additionally, these Piwi paralogs are differentially expressed in aphid reproductive morphs, suggesting that they may have specialized to function in different reproductive strategies (Lu et al. 2011).

Despite this diversity, much of our functional understanding of arthropod Argonautes comes from studies of *D. melanogaster* (Kataoka et al. 2001; Li et al. 2002; Pal-Bhadra et al. 2004; Vagin et al. 2004; Kalmykova et al. 2005; van Rij et al. 2006; Chung et al. 2008; Czech et al. 2008), which has two Ago-subfamily genes. Ago1 binds miRNAs and regulates gene expression by inhibiting translation of host transcripts (reviewed in Eulalio et al. 2008). Ago2 binds siRNAs from two sources: first, virus-derived small interfering RNAs, which guide Ago2 to cleave viruses or their transcripts, forming an integral part of the antiviral defense mechanism (Li et al. 2002; van Rij et al. 2006); second, endogenous siRNAs, which are derived from TEs, overlapping untranslated regions (UTRs) and other repetitive sequences in the soma (Chung et al. 2008; Czech et al. 2008). *Drosophila melanogaster* also encodes three Piwi-subfamily proteins, which bind piRNAs in the germline and surrounding tissues: Ago3, Aubergine (Aub), and Piwi (reviewed in Iwasaki et al. 2015). The piRNAs are differentiated from miRNAs and siRNAs in *D. melanogaster* by their Dicer-independent production and their amplification through the “Ping-Pong” pathway, a positive feedback loop involving Ago3 and Aub (Li et al. 2009). In *D. melanogaster*, piRNAs guide Piwi to TEs in euchromatin, where it inhibits transposition (Kalmykova et al. 2005) by directing the formation of heterochromatin (Sienski et al. 2012).

However, comprehensive analysis of Argonaute evolution at a eukaryotic, or even metazoan, scale is hindered by limited taxon sampling, wide variation in evolutionary rate, and the presence of ancient and recent duplications and losses (discussed by Philippe et al. 2011). The Diptera provide an opportunity to study Argonaute evolution in an order that is densely sampled and less divergent, but still shows variation in Argonaute copy number and function. Previous reports of Argonaute duplication in the Diptera have been limited to isolated taxa, such as the house fly *Musca domestica* (Scott et al. 2014), *Drosophila pseudoobscura* (Hain et al. 2010), and three mosquito species (Campbell et al. 2008). These mosquito duplicates appear to have evolved derived functions: Several Piwi paralogs in *Aedes aegypti* (Vodovar et al. 2012; Schnettler et al. 2013) and *Aedes albopictus* (Morazzani et al. 2012) are expressed in the soma, and at least one of the somatically expressed Piwi duplicates in *Ae.* aegypti appears to have functionally diverged to a novel antiviral function (Schnettler et al. 2013).

Gene duplications, such as those that gave rise to the diversity of eukaryotic RNAi pathways, are often associated with changes in evolutionary rate (reviewed in Hahn 2009), and *Drosophila* duplicates that evolve a new function often evolve more rapidly (Assis and Bachtrog 2013). However, the subsequent duration of this rate change can vary considerably, either changing only briefly following duplication (Nielsen et al. 2010), or persisting in all branches subtending the duplication event (Morandin et al. 2014). Additionally, rate change after duplication can be symmetrical or asymmetrical between the resulting paralogs: If both paralogs specialize to different pre-existing functions (subfunctionalization) they are expected to have roughly symmetrical evolutionary rates, whereas if one paralog undergoes neofunctionalization it is expected to evolve more rapidly than the other paralog, resulting in asymmetrical rates (Hittinger and Carroll 2007). Such a difference is seen after duplication of desaturase genes in *Drosophila*, which play key roles in evolutionary divergence and speciation through their contribution to the formation of cuticular hydrocarbons (Keays et al. 2011). These characteristic patterns of selection following duplication therefore enable us to use analyses of evolutionary rate to gain an insight into functional evolution.

Here we take advantage of the diversity available in the sequenced genomes and transcriptomes of Diptera to analyze patterns of Argonaute duplication and sequence evolution across 86 species. Contrasting rates of duplication and evolution are commonly associated with differences in function and selection pressure. We find a higher rate of protein evolution in Ago2 and Ago3, a higher rate of gene turnover in Ago2 and Piwi/Aub, and we estimate the date of the duplication that led to the separate Piwi and Aub subclades. We also find that paralogs of Ago2, Ago3, and Piwi/Aub evolve more rapidly after duplication, indicating potential divergence into novel and strongly selected functions.

## Materials and Methods

### Identification of Argonaute Homologs

We used TBLASTX and TBLASTN (Altschul et al. 1997) to identify Argonaute homologs in the genomes and transcriptomes of 86 Dipteran species found in GenBank, Flybase, Vectorbase, Diptex, the NCBI Transcriptome Shotgun Assembly, or other unpublished transcriptomes (see Supplementary Materials for a detailed list of sources; novel sequences have been submitted to GenBank as KR012647–KR012696). For each species, we used Argonautes from the closest well-annotated relative as queries, or *D. melanogaster* if no homolog from a close relative was available. Where BLAST returned multiple partial hits, we assigned hits to the correct query sequence by aligning all hits from the target species to all Argonautes from the query species, and inferring a neighbor-joining tree. For each query sequence, partial BLAST hits were then manually curated into complete genes using Geneious v5.6.2 (http://www.geneious.com/, last accessed April 15, 2012; Kearse et al. 2012). For some species of *Drosophila*, polymerase chain reaction (PCR) and Sanger sequencing was used as no transcriptomic or genomic data were available (see Supplementary Materials for details of genes).

### Phylogenetic Analysis of Dipteran Argonautes

We initially assigned homologs into subclades (Ago1, Ago2, Ago3, and Piwi/Aub) based on a Bayesian gene tree rooted between the Ago and Piwi subfamilies, with ambiguous alignment positions removed using Gblocks (Castresana 2000) and with the wasp *Nasonia vitripennis* as the outgroup for each subclade. We repeated this analysis with three other arthropod species as outgroup (*Bemisia tabaci*, *Bombyx mori*, and *Tribolium castaneum*), and found that in each case the same Dipteran genes were classified into the Ago1, Ago2, Ago3, and Piwi/Aub subclades. To minimize the loss of information when removing ambiguous positions, we reinferred separate Bayesian gene trees for each subclade with no outgroup, using new alignments with ambiguous positions identified by eye and removed (see Supplementary Materials for alignments). Sequences were aligned using translational MAFFT (Katoh et al. 2002) with default parameters. All phylogenies were inferred using the Bayesian approach implemented in MrBayes v3.2.1 (Ronquist and Huelsenbeck 2003) under a nucleotide model, assuming a general time reversible (GTR) substitution model with three unlinked codon-position classes, gamma-distributed rate variation between sites with no invariant sites, and inferred base frequencies. We ran each analysis for a minimum of 50 million steps, or as long as necessary for the tree topologies to reach stationarity (standard deviation of split frequencies between duplicate independent runs <0.01; potential scale reduction factor (PSRF)∼1 and effective sample size (ESS) > 1,000 for all parameters). Samples from the posterior were recorded every 10,000 steps, and a maximum clade credibility tree was inferred from 2 duplicate runs using TreeAnnotator (Drummond et al. 2012).

### Gene Turnover Rates

To quantify the rate of gene duplication and loss during Argonaute evolution, we estimated the rate of gene turnover (λ, the number of gains or losses per million years) for each Argonaute subclade using CAFE v3.1 (Han et al. 2013). We also tested whether subclades differed significantly in their rates of gene turnover by using 1,000 replicates of CAFE’s Monte Carlo resampling procedure. This generates an expected distribution of gene family sizes under a birth–death model, conditioned on the species topology and a set λ value (which we fixed at the value estimated for each subclade), thus providing an estimate of the *P* value for each of the other subclades. To mitigate potential bias introduced by incomplete genome assemblies, turnover analyses only included species that had at least one gene in each subclade (66 of total 86 species). To assess the potential impact of searching transcriptomes, which will only detect expressed genes (and may therefore lead to erroneous inference of gene loss and falsely inflate the rate of gene turnover), we repeated these analyses with rates of gene gain and loss estimated separately. We find similar results when comparing rates of gene gain and gene turnover, suggesting that missing data have a negligible effect on our estimates of gene turnover rate.

To provide the independent species-level tree topology for all 66 species that is required for this analysis, we manually combined the high-confidence multigene phylogenies presented in Wiegmann et al. (2011) and Misof et al. (2014). Where these reference trees lacked the relevant taxa (e.g., relationships below the level of family), we either referred to other published multigene phylogenies—van der Linde et al. (2010), Zhang et al. (2010), and Dyer et al. (2008) for Drosophilidae, *Bactrocera*, and *Glossina*, respectively—or inferred a Bayesian phylogeny using the arginine kinase gene (Culicidae, MrBayes parameters as above). Conditional on this species topology, we estimated relative branch lengths using BEAST v1.7 (Drummond et al. 2012) and a translational MAFFT alignment of the 1:1:1 ortholog Ago1, constraining the dates of key nodes to previously inferred dates derived from fossil evidence (as used by Wiegmann et al. 2011: Root = 245 Ma, Brachycera = 200 Ma, Cyclorrhapha = 150 Ma, Schizophora = 70 Ma). As our primary concern is the difference in relative rates of gene gain and loss for the different subclades, inaccuracies of the absolute timescale will have minimal impact on our conclusions.

### Evolutionary Rate and Positively Selected Residues

To infer the relative rates of synonymous and nonsynonymous substitution (d*N*/d*S* = ω) averaged across all sites, we used codeml (PAML; Yang 1997) to fit model M0 (single ω) separately for each subclade (Ago1, Ago2, Ago3, and Piwi/Aub), conditional on the alignment and tree topology. To test for significant differences between these subclade-specific rates, we fixed ω for each subclade at the value estimated for each of the other subclades, and used Akaike weights to compare the likelihood of these fixed ω values with the likelihood of the ω value estimated from the data for that subclade.

**Fig. 1.—:**
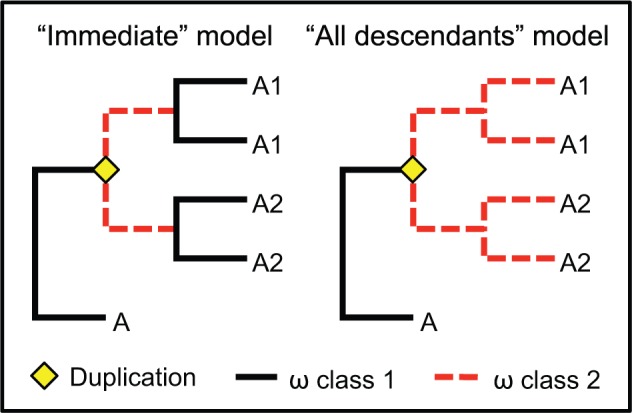
The two models fitted to branches after duplication events. Immediate models the expectation if selection pressures change only briefly after duplication, whereas All descendants models the expectation if paralogs evolve at a consistently different rate.

To estimate the change in evolutionary rate after duplication, and to test whether duplicates experienced a transient or sustained change in evolutionary rate, we fitted two variants of the M0 model, each with two separate ω parameters estimated for different branches of the gene tree (fig. 1). To test for a transient change in evolutionary rate directly after duplication, we fitted a model (which we term “Immediate”) that specified one ω for branches immediately after a duplication event, and another ω for all other branches. To test for a sustained change in evolutionary rate following duplication, we fitted a second model (which we term “All descendants”) that specified one ω for all branches arising from a duplication event, and another ω for all other branches. For each subclade, Akaike weights were used to estimate the relative support for the M0, Immediate, and All descendants models.

To test for asymmetrical evolutionary rates after a particular duplication event, we fitted two variants of the M0 model. The first model (which we term “Asymmetrical rates”) estimated three separate ω parameters for different branches of the gene tree: One ω for the branches in one lineage produced by the duplication event; a second ω for the branches in the other lineage; and a third ω for the rest of the tree. The second model (which we term “Symmetrical rates”) estimated two ω ratios: One ω for both lineages arising from the duplication event, and a second ω for the rest of the tree. The large number of nested duplication events means that an exhaustive test of all cases in which some duplication events result in asymmetrical rates is intractable; we therefore focused on ten key duplication events in the Ago2 and Piwi/Aub subclades (supplementary fig. S1, Supplementary Material online). For each duplication event, we used a likelihood ratio test (LRT) to compare the fit of the Asymmetrical and Symmetrical rates models. Following the method outlined above, we fitted Immediate and All descendants versions of each of these models.

To test for positively selected residues in each subclade, we used LRTs to compare the fit of two models, each with two site classes. In both models, ω of the first “constrained” site class was a discretized beta-distribution with eight classes. The models differ in that in the first model (the null model “M8a” in codeml) ω of the second “positively selected” site class is fixed at 1 (neutrality), while in the second model (the “M8” model) ω of the second site class is constrained to exceed 1. If the LRT indicated a significantly better fit for M8 than M8a given the parameters in the model, individual residues were classed as positively selected if they had a Bayes Empirical Bayes (BEB) posterior probability of >95% that ω > 1.

To assess the potential impact of false positives introduced by misalignments (Jordan and Goldman 2012), we ran M0 and M8 codeml analyses on two alignments for each subclade, the first with no trimming of ambiguous alignment positions (which may represent genuinely rapidly evolving sites), and the second with ambiguous alignment positions identified by eye and removed**.** All estimates and statistical comparisons of evolutionary rates outlined above were very similar with and without alignment screening: We therefore report results estimated from the untrimmed alignments. Although we could not rule out gene conversion between paralogs (which can lead to erroneous support for positive selection; Casola and Hahn 2009), we found very few positively selected sites, so this effect is likely to have little or no effect on our analyses.

### Domain Mapping and Structural Modeling

To investigate the distribution of rapidly evolving sites across the domain architecture of each Argonaute gene, we inferred the location of each domain in each Argonaute gene by searching the Pfam database (Finn et al. 2009), and then mapped the mean estimate of ω for each residue across the gene (derived from the BEB posterior distribution under the M8 model in PAML; Yang 1997). To describe evolutionary rate heterogeneity in the protein structures of each gene, we built structural models based on published X-ray crystallography structures: The *D. melanogaster* Ago1 structure was based on human Ago1 (Faehnle et al. 2013), and the structures of *D. melanogaster* Ago2, Ago3, and Piwi were based on human Ago2 (Schirle and Macrae 2012). We used the MODELER software in the Discovery Studio 4.0 Modeling Environment (Accelrys Software, Inc., San Diego) to calculate ten models, and selected the most energetically favorable for each protein. The model optimization level was set to High, and loop refinement was included. The model quality was assessed with the three-dimensional (3D) profile option in the software, which compares the compatibility of the 3D structure and the sequence. For *D. melanogaster* Ago2, we replaced the inferred PAZ domain structure with the *D. melanogaster* Ago2 PAZ domain structure that has previously been resolved using X-ray crystallography (Song et al. 2003). We then mapped ω onto each residue of the structure using PyMol v.1.7.4.1 (Schrödinger, LLC). For both analyses, we used estimates of ω from trimmed alignments to provide a conservative estimate of residue-specific evolutionary rate. Sites that were trimmed out of the alignment were excluded when mapping ω across domains, and were set as ω = 0 when mapping ω across structures.

## Results

### Duplications of Ago2, Ago3, and Piwi/Aub Occur in Different Dipteran Lineages

**Fig. 2.—:**
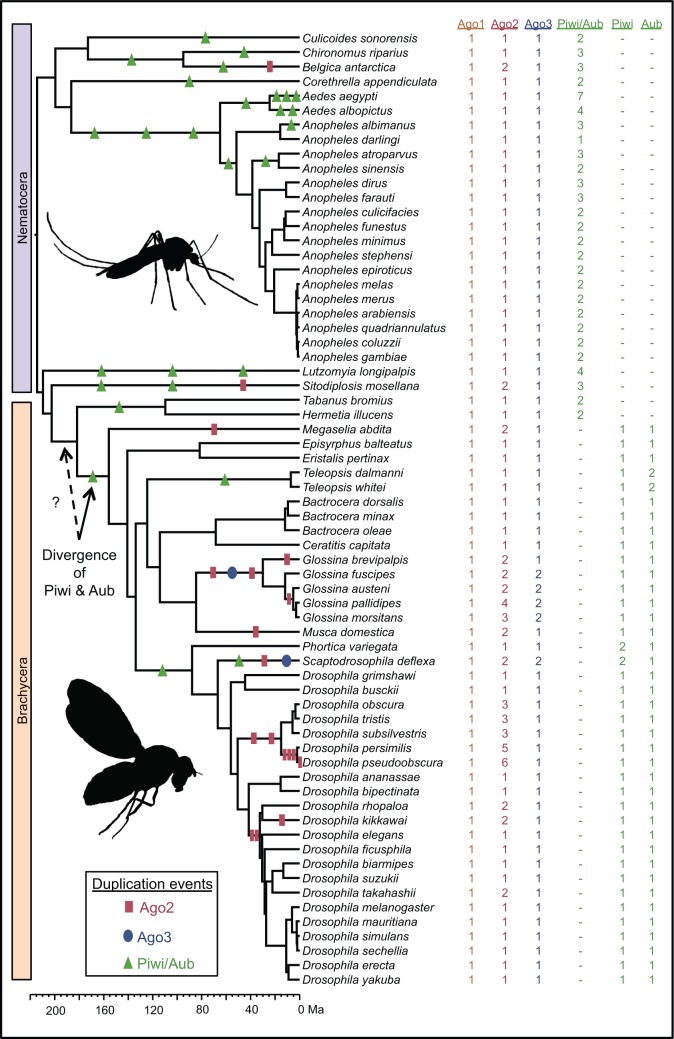
Counts of each Argonaute subclade. Shown are counts for a subsample of 66 Dipteran species with at least one gene in each subclade (out of a total of 86 species). Gene duplication events were inferred by parsimony, and are illustrative only (gene loss is not depicted due to space constraints, thus for some taxa gene counts do not correspond to the number of gene duplications). The rate of gene turnover differs between different Argonautes and lineages, and the divergence of Piwi and Aub occurred 182–156 Ma. Silhuoettes by Warren Photographic and Ramiro Morales-Hojas.

**Fig. 3.—:**
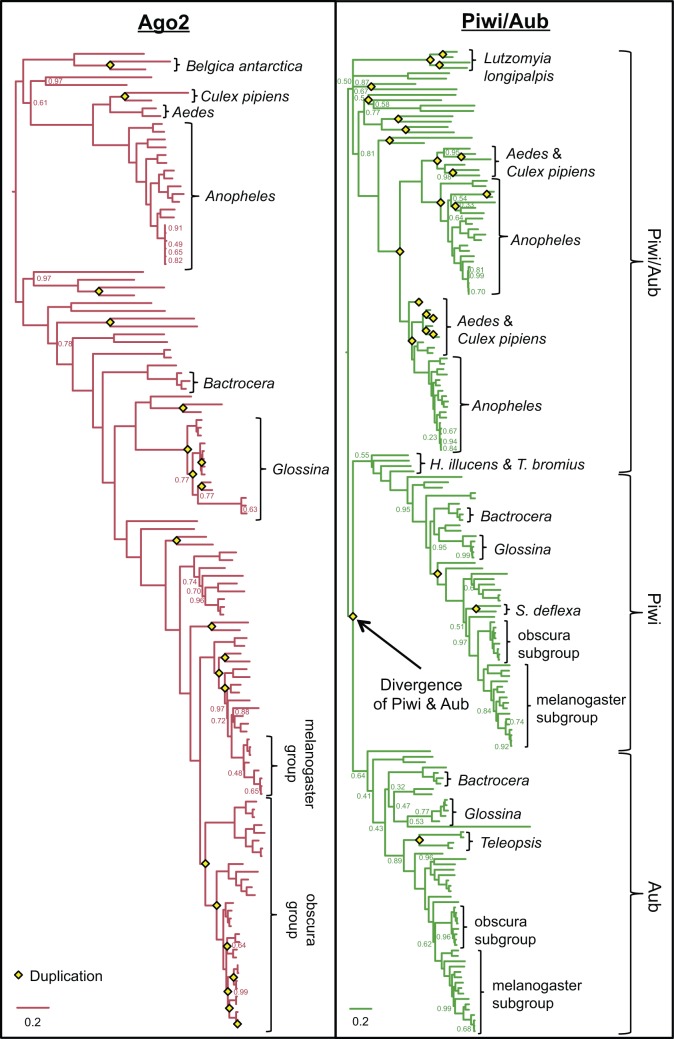
Bayesian gene trees of Ago2 and Piwi/Aub. Ago2 has expanded rapidly in *Glossina* and the obscura group of *Drosophila*, whereas Piwi/Aub has undergone numerous duplications in *Aedes*, *Anopheles*, and many other Nematoceran taxa.

To explore the evolutionary dynamics of Argonautes in the Diptera, we quantified the rate of duplication and evolution of Argonautes from 86 Dipteran species. We find numerous expansions of Ago2 and Piwi/Aub (including the origin of canonical Piwi and Aub themselves from their Piwi-subfamily ancestor; figs. 2 and 3). This is in sharp contrast to Ago1, which is present as a single copy ortholog in all Diptera (fig. 2 and supplementary fig. S2, Supplementary Material online), and Ago3, which has duplicated only rarely (fig. 2 and supplementary fig. S3, Supplementary Material online).

We also find that the expansions of Ago2 and Piwi/Aub have occurred in different taxa and at different times (figs. 2 and 3). Most duplications of Ago2 have occurred in the Brachycera, with numerous duplications within the *Glossina* (<84 Ma), and the *Drosophila obscura* group (<50 Ma) (Hain et al. 2010). Perhaps surprisingly, even in the melanogaster group there appear to have been at least three duplications: A duplicate shared between *Drosophila rhopaloa* and *Drosophila takahashii* (DRHO009538 and DTAK011769, respectively) implying multiple losses, and a duplication within the lineage leading to *Drosophila kikkawai*. Although incomplete genomes and some uncertainty in the gene tree topology mean that the losses are uncertain, implications for our study are minimal, as our analysis of gene turnover uses only gene counts, and losses do not factor into our comparison of evolutionary rates before and after duplication. Single duplications of Ago2 have occurred in the Brachycerans *Drosophila willistoni*, *Scaptodrosophila deflexa*, *M. domestica*, and *Megaselia abdita*, and in the Nematocerans *Belgica antarctica*, *Culex pipiens*, and *Sitodiplosis mosellana* (fig. 3).

In contrast, most duplications of Piwi/Aub have occurred in the Nematocera. Numerous duplications have occurred in the mosquitoes (*Aedes* spp., *Anopheles* spp., and *Culex quinquefasciatus*) <65 Ma, and multiple copies are seen in *Lutzomyia longipalpis*, *Si. mosellana*, *Chironomus riparius*, *B. antarctica*, and *Corethrella appendiculata* (fig. 3). A duplication at the base of the Brachycera between 182 and 156 Ma gave rise to the separate Aub and Piwi subclades (as they occur in *D. melanogaster*, labeled in figs. 2 and 3). Within these subclades duplications have occurred rarely, only being observed in Piwi of the drosophilids *Phortica variegata* and *Sc. deflexa*, and in Aub of *Teleopsis* species.

### Ago2 and Piwi/Aub Have Significantly Higher Duplication Rates than Ago1 and Ago3

To quantify these contrasting patterns of duplication, we used CAFE (Han et al. 2013) to estimate the rate of gene turnover (λ, the number of gains or losses per million years) in each Argonaute subclade. We find that gene turnover rate varies considerably among the subclades, with Ago2 (λ = 0.0022), Ago3 (λ = 0.0003), and Piwi/Aub (λ = 0.0012) having significantly higher gene turnover rates than Ago1 (λ = 1.1516 × 10^−^^10^) (*P* < 0.001 based on the expected distribution of gene family sizes under a birth–death model, with λ fixed at the value estimated for Ago1). We also find that Ago2 and Piwi/Aub have significantly (*P* < 0.001) higher gene turnover rates than Ago3, but do not differ significantly from each other (*P* = 0.198).

### Argonautes Show Contrasting Rates of Protein Evolution Before and After Duplication

To quantify the rate of protein evolution in each Argonaute subclade, and to identify any sites evolving under positive selection, we fitted models using codeml (PAML; Yang 1997). These analyses revealed that Ago2 has the highest nonsynonymous to synonymous substitution ratio (ω = 0.14 ± 0.0015), followed by Ago3 (ω = 0.12 ± 0.0015), Piwi/Aub (ω = 0.09 ± 0.0009), and finally Ago1 (ω = 0.01 ± 0.0002). All rates were significantly different from each other (Akaike weight = 1.000 to 3 decimal places (d.p.) in all cases). Scans for positively selected sites identified five candidate sites in Ago3 and one in Piwi/Aub; however, in both cases the M8 model was not significantly more likely than the null M8a model (for ω estimates and likelihoods under all models, see supplementary tables 1–3, Supplementary Material online).

**Fig. 4.—:**
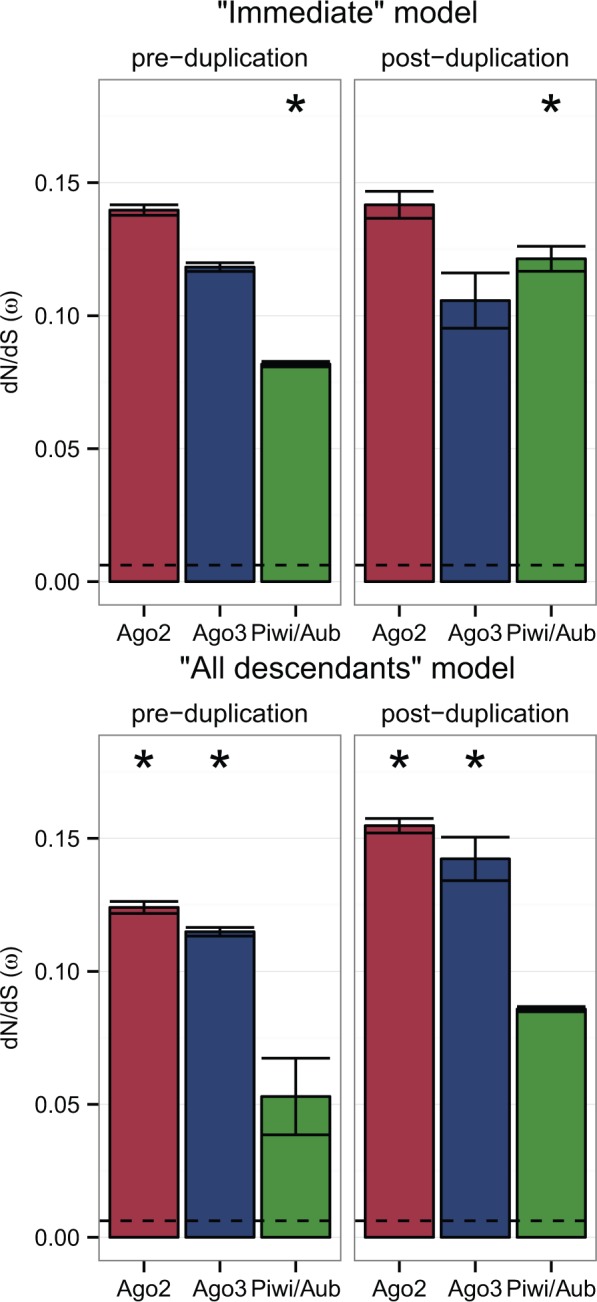
Evolutionary rate estimates before and after duplication, under the Immediate and All descendants models. Asterisks indicate the most highly supported model, and the dashed line indicates the ω value for Ago1 under the M0 model. Duplicates of Piwi/Aub evolve more quickly immediately after duplication, whereas Ago2 and Ago3 paralogs experience a sustained increase in evolutionary rate.

To test whether the relative rate of protein evolution changes following duplication, we calculated the likelihood of the data for Ago2, Ago3, and Piwi/Aub under two models: The first with a separate evolutionary rate for branches immediately after a duplication event (the Immediate model); and the second with a separate rate for all branches subtending a duplication event (the All descendants model) (fig. 1). For Ago2 and Ago3, the All descendants model had all support (Akaike weight = 1.000 to 3 d.p. for each). For Piwi/Aub, however, the Immediate model had all support (Akaike weight = 1.000 to 3 d.p.). In each case the evolutionary rate increased after duplication, with Ago2 having the highest rate and Piwi the lowest (fig. 4).

To test for asymmetry between the evolutionary rates of paralogs after duplication, we calculated the likelihood of the data for ten key duplication events in the Ago2 and Piwi/Aub subclades under two models: The Asymmetrical rates model specified one evolutionary rate for one lineage produced by the duplication event, a second rate for the other lineage, and a third for the rest of the tree; and the Asymmetrical rates model specified one evolutionary rate for both lineages produced by the duplication event, and a second rate for the rest of the tree. For Ago2, we find that the Asymmetrical rates model does not provide a significantly better fit for the *Glossina* sp., *B. antarctica*, or *Cu. pipiens* duplication events (LRT, *P* > 0.1 in all cases). However, the Asymmetrical rates model fits significantly better for the two branches immediately after the *obscura* group Ago2e-Ago2a/f event (LRT, *P* < 0.005), and for all branches subtending this event (LRT, *P* < 0.005). Under the Asymmetrical rates (All descendants) model, the Ago2e clade (ω = 0.17 ± 0.011) and Ago2a/Ago2f clade (ω = 0.22 ± 0.009) are evolving considerably faster than the rest of the tree (ω = 0.13 ± 0.001). For the *obscura* group Ago2a-Ago2f event, the Asymmetrical rates (All descendants) model does not provide a significantly better fit (LRT, *P* > 0.1), but the Immediate version of this model does give a significantly better fit (LRT, *P* < 0.005). Under this model, the branch at the base of the Ago2a clade has a much lower evolutionary rate (ω = 0.06 ± 0.029) than the rest of the tree (ω = 0.14 ± 0.001), and the branch at the base of the Ago2f clade has a much higher evolutionary rate (ω = 0.42 ± 0.142).

For Piwi/Aub, we find that the Asymmetrical rates (All descendants) model provides a significantly better fit for the Piwi–Aub divergence (LRT, *P* < 0.01) and the duplication event early in mosquito evolution (LRT, *P* < 0.005). The Aub lineage (ω = 0.09 + 0.002) has a higher evolutionary rate than the Piwi lineage (ω = 0.08 ± 0.002); however, the similarity between the rate of Aub and the rest of the tree (ω = 0.09 ± 0.002) suggests that this difference may be caused by constraint on Piwi rather than positive selection on Aub. After the mosquito Piwi/Aub duplication event, we see a large difference in evolutionary rates, with one clade evolving much more rapidly (ω = 0.14 ± 0.004) than the other (ω = 0.04 ± 0.003), and the rest of the tree (ω = 0.08 ± 0.001).

### Ago2 Displays Hotspots of Evolution at the RNA Binding Pocket Entrance

**Fig. 5.—:**
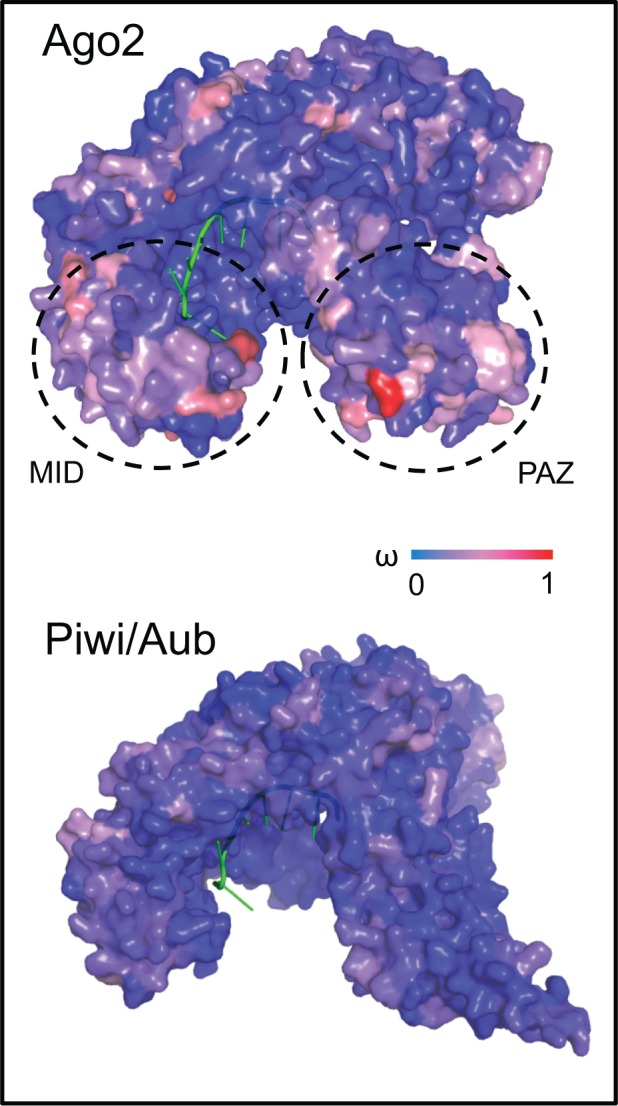
Evolutionary rates mapped onto 3D structures of Ago2 and Piwi/Aub, each binding a sRNA guide. In Ago2, hotspots of evolution are seen at the entrance of the RNA binding pocket; in contrast, evolutionary rate (ω) across the structure of Piwi/Aub is uniformly low. The MID and PAZ protein domains are indicated for Ago2.

To investigate the distribution of rapidly evolving residues, we mapped ω estimates onto the domains and structures of each Argonaute. We found that rapidly evolving residues are spread across all domains of Ago2, Ago3, and Piwi/Aub (supplementary fig. S4, Supplementary Material online). We also found that Ago2 appears to have clusters of more rapidly evolving residues at the entrance to the RNA binding pocket (fig. 5), which are not found in Ago3 (supplementary fig. S5, Supplementary Material online) or Piwi/Aub (fig. 5). In contrast, the residues that directly contact the sRNA guide are conserved in all Argonautes (fig. 5 and supplementary fig. S5, Supplementary Material online).

## Discussion

Our results reveal contrasting patterns of selection and duplication during Dipteran Argonaute evolution. The low evolutionary rate and lack of gene turnover in Ago1 are in agreement with previous studies in *D. melanogaster* (Obbard et al. 2006, 2009), and are consistent with the idea that Ago1 is carrying out a conserved gene regulatory role in the Diptera as a whole. In contrast, the better fit of the All descendants model to duplications in Ago2 and Ago3 (fig. 4) indicates that paralogs in these subclades have experienced a sustained increase in evolutionary rate, possibly driven by the acquisition of new functions.

This result is particularly noteworthy in Ago2, which is already among the top 3% of the fastest evolving proteins in *D. melanogaster* (Obbard et al. 2006). Our structural modeling suggests that one possible hotspot of adaptive evolution for these paralogs may be the entrance of the RNA binding pocket (fig. 5). Relaxation of selection pressures on these residues is unexpected as they form alpha-helices, rigid secondary structures that are needed for the stability of the tertiary structure of the protein (Panchenko et al. 2005; Liu et al. 2008); instead, their rapid evolution may be caused by undetected positive selection. The pocket is formed by the PAZ and MID domains, which bind the sRNA guide and form the channel in which the target RNA sits during cleavage (Schirle and Macrae 2012). Although the molecular interactions between the sRNA guide and the inside of the binding pocket have been characterized (reviewed in Swarts et al. 2014), less is known about the function of the residues at the entrance to the pocket. However, given the location of these rapidly evolving residues at the mouth of the binding pocket away from the sRNA guide (fig. 5), such positive selection could be driving differences in target RNA binding and cleavage. Alternatively, selection could be imposed by viral suppressors of RNAi, which are encoded by numerous viruses to inhibit the antiviral RNAi response, and several of which prevent target cleavage by Ago2 (Wang et al. 2006; van Mierlo et al. 2012, 2014). Although we do not find evidence of positive selection in our site analysis across the Diptera as a whole, signatures of selection are evident when we apply branch-sites analyses to Ago2 in the Drosophilidae, as has been reported previously (Kolaczkowski et al. 2011). Such selection may be acting on Ago2 in the Diptera as a whole, but its signature may be masked by saturation of synonymous sites (Anisimova et al. 2001; Clark et al. 2007).

Functional differences between most Dipteran Argonaute paralogs have not been characterized experimentally. However, transcriptome data are available for some *G. morsitans* tissues, including “lactating” and nonlactating females (Benoit et al. 2014) and salivary glands from parasitized and unparasitized individuals (Telleria et al. 2014) (SRA accessions SRX287393, SRX287395, SRX342351, and SRX342350, respectively). Using these data we explored the possibility of functional divergence in *G. morsitans* Ago2 and Ago3 paralogs, and found differential expression between both sets of paralogs, as well as high expression of Ago3b in the salivary glands, which increased upon infection with *Trypanosoma brucei* (supplementary fig. S6, Supplementary Material online). Although this observation awaits replication, the canonical germline-specific role of Ago3 in *D. melanogaster* (Li et al. 2009) makes any expression of *G. morsitans* Ago3b in the salivary glands unexpected, and suggests that this paralog has undergone rapid functional divergence to a role beyond TE suppression. Strikingly, this reflects the general patterns noted for somatically expressed Piwis across the eukaryotes, which have evolved diverse roles in epigenetic regulation, genome rearrangement, and somatic development (reviewed in Ross et al. 2014).

The better fit of the Immediate model to duplications of Piwi/Aub (fig. 4) suggests that the evolutionary rate of paralogs in these subclades has been constrained soon after duplication, which may indicate a burst of adaptation to specialize to existing (but distinct) roles. For many duplicates, the branches immediately after duplication are also terminal branches, which clouds the difference between the Immediate and All descendants models. In contrast, the divergence of separate Aub and Piwi (sensu stricto) lineages resulted from a much older duplication in the Piwi-subfamily Piwi/Aub subclade. Our asymmetry analysis suggests that this divergence was accompanied by a reduction in evolutionary rate, particularly in the Piwi lineage, indicating that these lineages are evolving under tight constraint. We estimate that this divergence, which happened at the base of the Brachycera, occurred between 182 and 156 Ma (fig. 2). However, the ambiguous identities of the two Piwi/Aub paralogs in *Hermetia illucens* and *Tabanus bromius* (fig. 3) mean that this duplication could have occurred slightly earlier (∼200 Ma).

Under either scenarios, Piwi/Aub paralogs in the vast majority of Nematoceran taxa (including all mosquitoes) are equally homologous to Aub and Piwi, which in *D. melanogaster* have specialized to distinct roles in the Ping-Pong piRNA amplification cycle and TE silencing, respectively, suggesting that the ancestral Piwi/Aub gene may have had multiple conflicting functions (reviewed in Luteijn and Ketting 2013). It may be that the increased duplication rate of Piwi/Aub in the Nematocera is a result of multiple independent resolutions of this conflict, causing piRNA biogenesis and TE silencing to rely on different suites of Argonaute genes in the Nematocera. This is supported by our asymmetry analysis, which finds that the Piwi/Aub expansion in mosquitoes resulted in asymmetrical evolutionary rates in the resulting lineages, with the rapid evolution of one lineage consistent with the evolution of a novel function. Notably, this rapidly evolving lineage includes a Piwi/Aub paralog in *Ae. aegypti* (Piwi5) that has recently been shown to have a highly derived function in the production of virus-derived piRNAs (Miesen et al. 2015). *Aedes aegypti* is a major vector of several arboviruses including yellow fever virus and chikungunya virus, and also has an exceptionally high TE load (Arensburger et al. 2011); although little is known about the total viral load of *Ae. aegypti*, it is possible that the combined viral and TE loads impose contrasting selection pressures, thereby driving the expansion of Piwi/Aub. Moreover, the numerous instances of expansion followed by functional divergence demonstrate that the Piwi/Aub subclade is not constrained to a germline-specific anti-TE role, but can evolve novel and highly derived functions.

## Conclusion

We show that Dipteran Argonautes differ widely in their rates of gene turnover and protein evolution, with duplication driving an increase in evolutionary rate that suggests frequent functional divergence. Our results provide an insight into the selection pressures driving the evolution of RNAi mechanisms across the eukaryotes, which are integral to a range of cellular and genomic processes. Our finding that Argonautes undergo frequent expansions and contractions indicates that expansions in other taxa, such as the WAGO clade of nematodes (Buck and Blaxter 2013) and the Piwi clade of the aphid *Ac. pisum* (Lu et al. 2011), are not isolated cases; instead, these are further examples of a general pattern of rapid gene turnover in some Argonaute clades. Additionally, our finding that duplication drives rapid evolution suggests that Argonautes evolve new functions frequently and rapidly, as exemplified by Ago4 in the shrimp *Penaeus monodon* (Leebonoi et al. 2015) and the smedwi clade of planarians (Reddien et al. 2005). This combination of rapid gene turnover and frequent functional divergence illustrates a high degree of evolutionary lability in Argonaute function across a wide range of taxa, and may drive the functional overlap frequently observed between different Argonaute subclades across the eukaryotes. Our work also highlights the selection pressures exerted by parasites, shown by the higher rate of gene turnover for Piwi/Aub and higher evolutionary rate of Ago2, which play roles in defense against TEs and viruses, respectively. This provides further evidence of the importance of parasites in evolution (Dawkins and Krebs 1979), and demonstrates how host–parasite interactions can drive genome evolution and generate phenotypic novelty.

## Supplementary Material

Supplementary tables S1–S3 and figures S1–S6 are available at *Genome Biology and Evolution online* (http://www.gbe.oxfordjournals.org/).

Supplementary Data
