# Supplementary material for: Duplication and Diversification of Dipteran Argonaute Genes, and the Evolutionary Divergence of Piwi and Aubergine
Source: Genome Biol Evol. 2016 Feb 11;8(3):507–18. doi: 10.1093/gbe/evw018 (PMC4824172; doi:10.1093/gbe/evw018)
Supplement: Supplementary Data [file supp_8_3_507__index.html]

Duplication and Diversification of Dipteran Argonaute Genes, and the Evolutionary Divergence of Piwi and Aubergine — Supplementary Data 

# Duplication and Diversification of Dipteran Argonaute Genes, and the Evolutionary Divergence of Piwi and Aubergine

## Supplementary Data

files

- Supplementary Data - zip file
