# Supplementary material for: Duplication and Diversification of Dipteran Argonaute Genes, and the Evolutionary Divergence of Piwi and Aubergine
Source: Genome Biol Evol. 2016 Feb 11;8(3):507–18. doi: 10.1093/gbe/evw018 (PMC4824172; doi:10.1093/gbe/evw018)
Supplement: Supplementary Data [file supp_evw018_suppl_data.zip › Lewis et al Supplementary resubmitted.pdf]

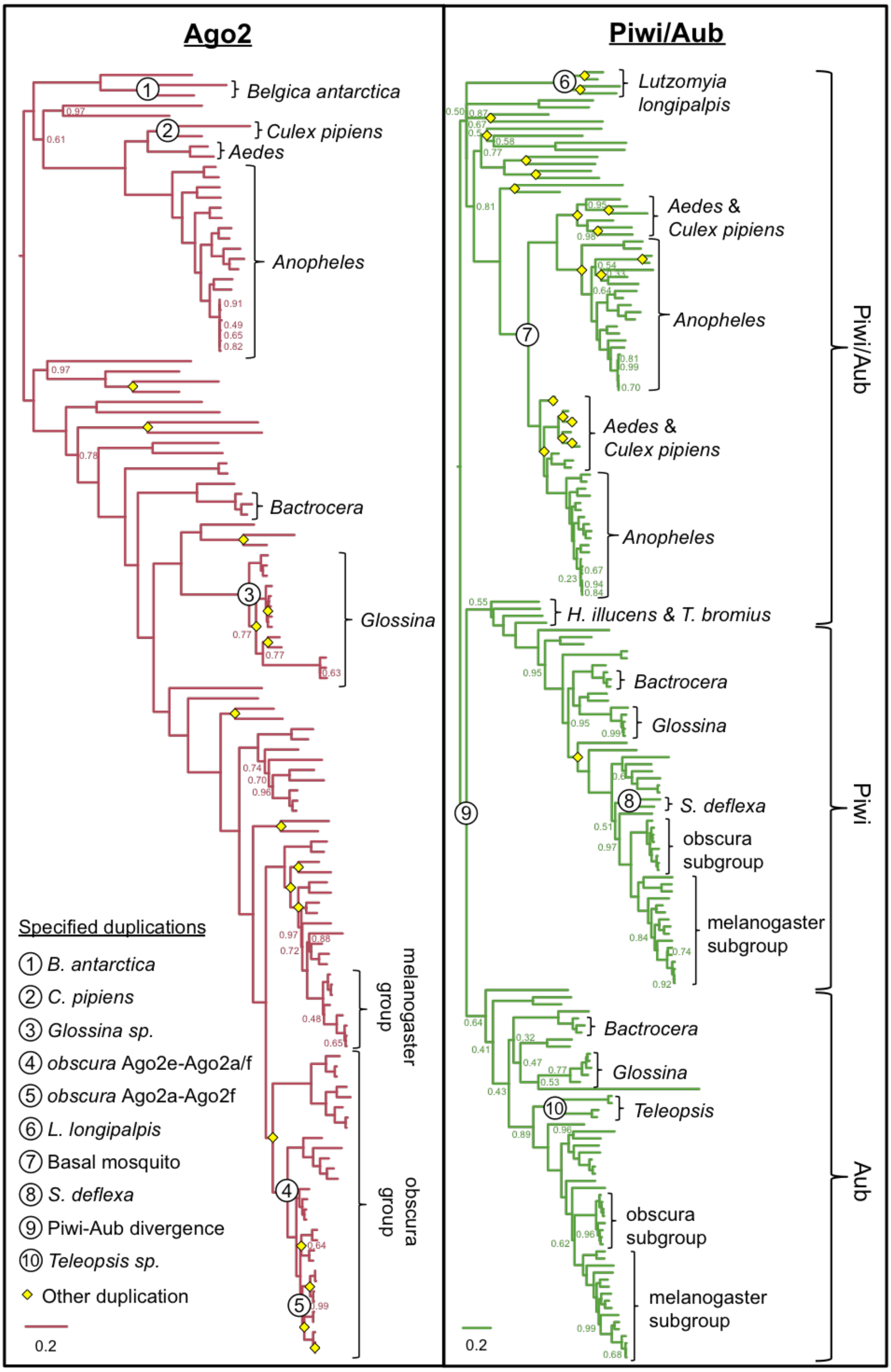

Figure S1: Duplication events specified in Asymmetrical rates and Symmetrical rates models. For each duplication event, a model estimating two separate rates for the two lineages after duplication (Asymmetrical rates) was compared with a model estimating one rate for both lineages (Symmetrical rates).

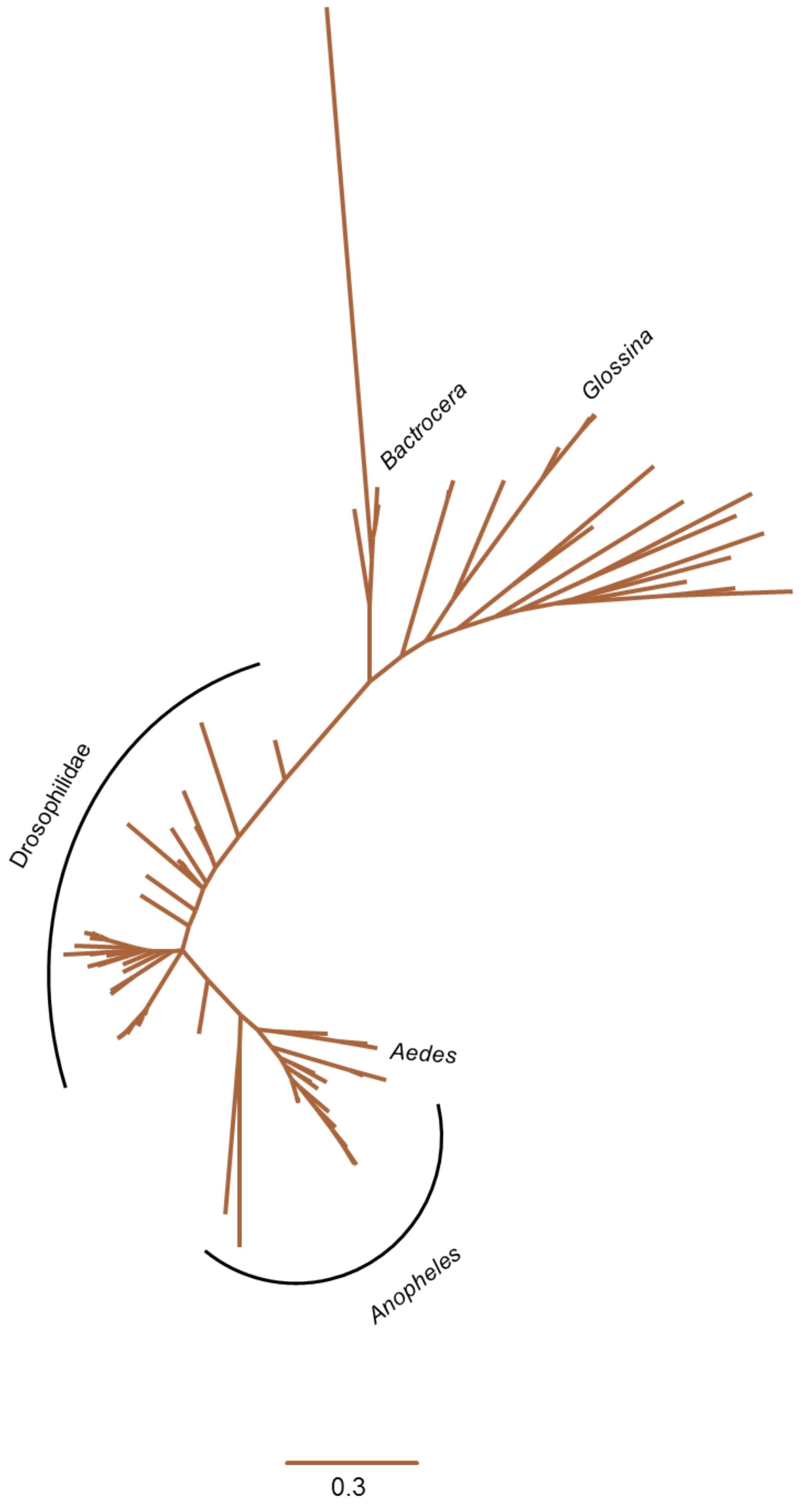

Figure S2: Unrooted Bayesian gene tree of Dipteran Ago1.
Ago1 has not duplicated and evolves very slowly in the Diptera, resulting in a lack of information that introduces some incongruence between the gene and species trees.

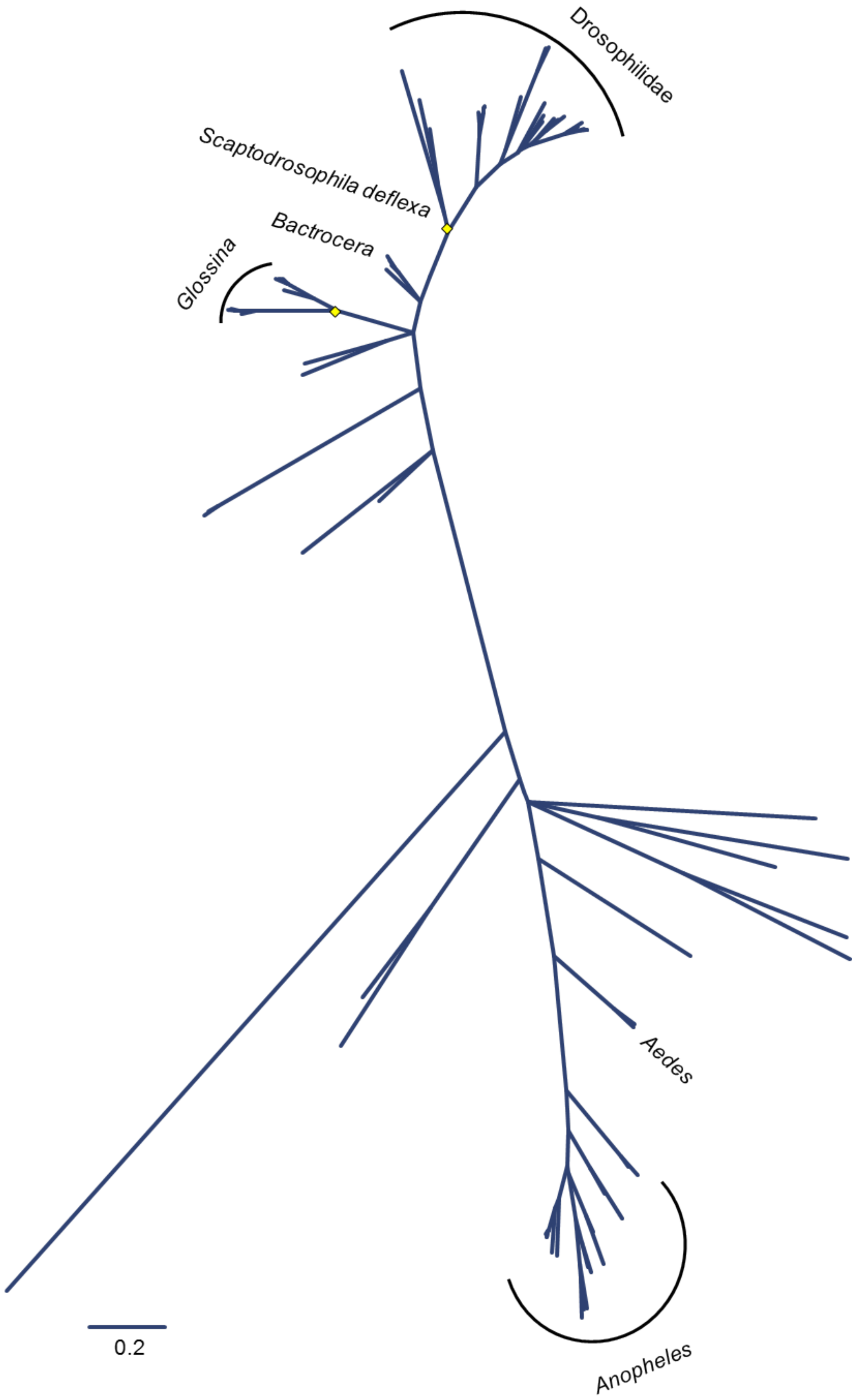

Figure S3: Unrooted Bayesian gene tree of Dipteran Ago3.
Ago3 has duplicated twice in the Diptera, once in *Scaptodrosophila deflexa* and once at the base of the *Glossina* species.

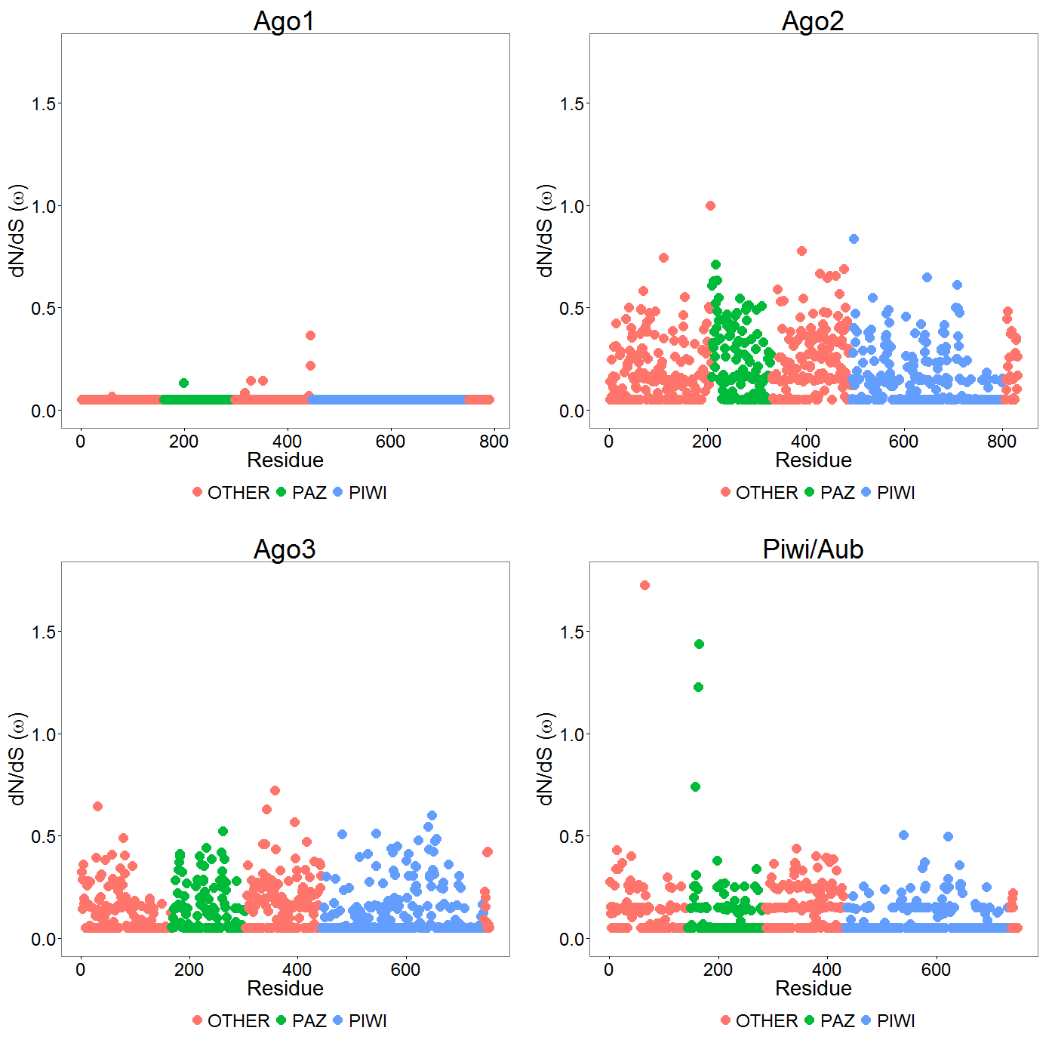

Figure S4: Evolutionary rates mapped onto the domain architecture of Dipteran Argonautes.
In each gene, rapidly evolving residues do not cluster in a particular location, but instead are spread across all domains.

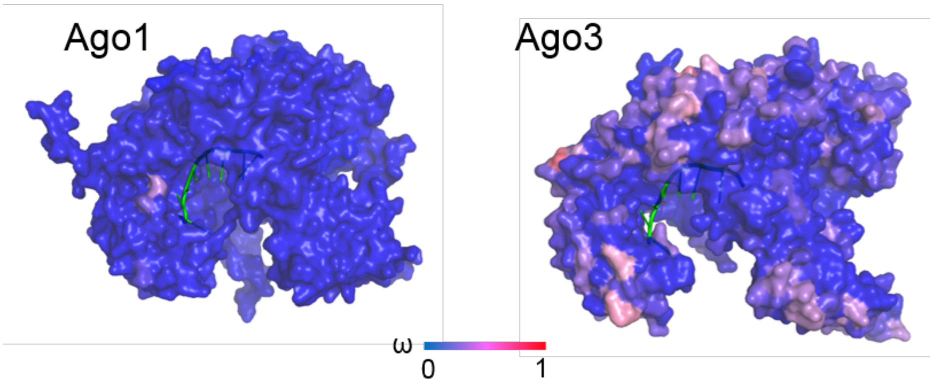

Figure S5: Evolutionary rates mapped onto the protein structures of Ago1 and Ago3.
In contrast to Ago2 (Figure 5), Ago1 and Ago3 do not show hotspots of evolution at the RNA binding pocket; instead, rapidly evolving residues are spread across the structure (Ago3) or absent (Ago1).

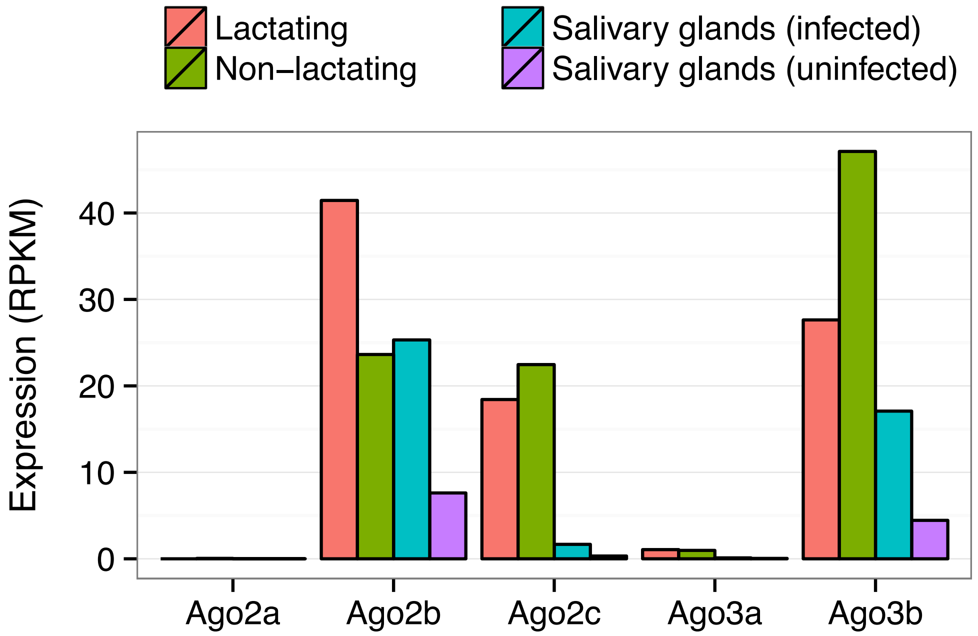

Figure S6: Tissue-specific expression patterns of paralogues of Ago2 and Ago3 in *G. morsitans*. Paralogues of Ago2 and Ago3 show divergent patterns of expression: this is particularly unexpected for Ago3b, which is expressed in the salivary glands of *G. morsitans*, despite *D. melanogaster* Ago3 being germline-specific.

Subclade	Model	lnL (2 d.p.)	ω (3 d.p.)
Ago1	M0	-48132.17	0.006 (±0.0002)
Ago1	M0 (ω fixed at Ago2 value)	-52340.60	0.140
Ago1	M0 (ω fixed at Ago3 value)	-51700.12	0.118
Ago1	M0 (ω fixed at Piwi/Aub value)	-50702.80	0.086
Ago2	M0	-150300.65	0.140 (±0.0015)
Ago2	M0 (ω fixed at Ago1 value)	-160382.25	0.006
Ago2	M0 (ω fixed at Ago3 value)	-150365.20	0.118
Ago2	M0 (ω fixed at Piwi/Aub value)	-150784.74	0.086
Ago3	M0	-90817.41	0.118 (±0.0015)
Ago3	M0 (ω fixed at Ago1 value)	-95795.68	0.006
Ago3	M0 (ω fixed at Ago2 value)	-90860.30	0.140
Ago3	M0 (ω fixed at Piwi/Aub value)	-90948.07	0.086
Piwi/Aub	M0	-194367.89	0.086 (±0.0010)
Piwi/Aub	M0 (ω fixed at Ago1 value)	-201332.12	0.006
Piwi/Aub	M0 (ω fixed at Ago2 value)	-195019.63	0.140
Piwi/Aub	M0 (ω fixed at Ago3 value)	-194629.29	0.118

Table S1: Log likelihood values and ω estimates for M0 (branch) models, comparing ω estimates between subclades.

Subclade	Model	lnL (2 d.p.)	ω (3 d.p.) (post-duplication)	ω (3 d.p.) (pre-duplication)
Ago2	"Immediate"	-150300.58	0.142 (±0.0051)	0.139 (±0.0020)
Ago2	"All descendants"	-150275.30	0.155 (±0.0027)	0.124 (±0.0023)
Ago3	"Immediate"	-90816.75	0.106 (±0.0104)	0.118 (±0.0016)
Ago3	"All descendants"	-90811.18	0.142 (±0.0082)	0.115 (±0.0016)
Piwi/Aub	"Immediate"	-194334.11	0.116 (±0.0045)	0.082 (±0.0011)
Piwi/Aub	"All descendants"	-194364.84	0.086 (±0.0010)	0.053 (±0.0143)

Table S2: Log likelihood values and ω estimates for M0 models with a separate ω for branches immediately after duplication ("Immediate"), or a separate ω for all branches subtending a duplication ("All descendants").

Subclade	Model	lnL (2 d.p.)
Ago1	M8	-47761.76
Ago1	M8a	-47759.73
Ago2	M8	-144338.35
Ago2	M8a	-144338.35
Ago3	M8	-87230.63
Ago3	M8a	-87206.97
Piwi/Aub	M8	-188055.00
Piwi/Aub	M8a	-188055.14

Table S3: Log likelihood values for M8 and M8a (sites) models.
